# Perceptual and Interoceptive Strength Norms for 270 French Words

**DOI:** 10.3389/fpsyg.2021.667271

**Published:** 2021-06-11

**Authors:** Aurélie Miceli, Erika Wauthia, Laurent Lefebvre, Laurence Ris, Isabelle Simoes Loureiro

**Affiliations:** ^1^Cognitive Psychology and Neuropsychology Department, Faculty of Psychology and Educational Sciences, University of Mons, Mons, Belgium; ^2^Institute of Health Sciences and Technologies, University of Mons, Mons, Belgium; ^3^Interdisciplinary Research Center of Psychophysiology and Cognitive Electrophysiology, University of Mons, Mons, Belgium; ^4^Neuroscience Department, Faculty of Medicine and Pharmacy, University of Mons, Mons, Belgium

**Keywords:** perceptual strength, interoception conceptualization, norms, semantic, psycholinguistic variables

## Abstract

Perceptual experience through the five modalities (i.e., vision, hearing, touch, taste, and smell) has demonstrated its key role in semantics. Researchers also highlighted the role of interoceptive information in the grounded representation of concepts. However, to this day, there is no available data across these modalities in the French language. Therefore, the aim of this study was to circumvent this caveat. Participants aged between 18 and 50 completed an online survey in which we recorded scores of perceptual strength (PS), interoceptive information, imageability, concreteness, conceptual familiarity, and age of acquisition of 270 words of the French language. We also analysed the relationships between perceptual modalities and psycholinguistic variables. Results showed that vast majority of concepts were visually-dominant. Correlation analyses revealed that the five PS variables were strongly correlated with imageability, concreteness, and conceptual familiarity and highlight that PS variables index one aspect of the semantic representations of a word. On the other hand, high interoceptive scores were highlighted only for the less imageable and less concrete words, emphasizing their importance for the grounding of abstract concepts. Future research could use these norms in the investigation of the role of perceptual experience in the representation of concepts and their impact on word processing.

## Introduction

Traditional conceptualizations of semantic knowledge mainly focused on the investigation of how concepts are organised in memory, notably by analysing relationships between them (Quillian, 1967; Collins and Loftus, 1975) or by evaluating their shared and distinct features (Farah and McClelland, 1991; McRae et al., 1997; Tyler and Moss, 2001; Vigliocco et al., 2004; Rogers et al., 2006). According to the classical neuropsychological approaches, conceptual knowledge would be symbolic and concepts would be stored in memory in an abstract way, independently of our sensorial modalities (Murphy, 2002). These models also posited that sensory and emotional information would only be considered during pre- and post-semantic processing steps (Fodor, 1983; Pexman, 2019). Over the past two decades, new conceptualizations emerged and progressively adopted an embodied approach which postulates that knowledge and conceptual representations are a direct result of interactions with the environment and are grounded in action and perception (Barsalou, 1999, 2008; Wilson, 2002). In this context, a growing number of studies observed that semantic representations rely on multimodal experiences and are associated with activations in several brain regions, including the sensory and motor cortex (e.g., González et al., 2006; Simmons et al., 2007; Kiefer et al., 2008; Fernandino et al., 2016; for reviews, see Thompson-Schill, 2003; Martin, 2007; Patterson et al., 2007; Binder, 2016).

The investigation of the conceptual involvement in semantic memory requires appropriate stimuli. In this perspective, norms have been developed to capture the degree to which a word evokes a sensory and/or perceptual experience in the participant’s mind (Juhasz et al., 2011; Juhasz and Yap, 2013; Bonin et al., 2015). In these studies, participants were asked to rate on a Likert scale the degree to which any given word evokes a sensory experience in a single note. This method has been shown to lead to information loss because the score did not refer to a specific perceptual modality. Therefore, auditory, haptic, and gustatory modalities were neglected by people while olfactory and visual information was distorted (Connell and Lynott, 2016). Consequently, Lynott and Connell (2009, 2013) have extended these first norms by asking participants to rate the extent to which they experience a word in each of the five sensory modalities on a scale of 0 (not experienced at all) to 5 (highly experienced). In these studies, they highlighted the importance of separately assessing perceptual modalities when trying to standardize the sensory bases of words and concepts in order to capture effects that are related to particular modalities and not to others (Connell and Lynott, 2012, 2016). For example, it has been shown that perceptual and conceptual processing share a disadvantage in the tactile modality relative to other perceptual modalities; people are slower and less accurate in detecting stimuli related to tactile sensations (Spence et al., 2001; Turatto et al., 2004) and are equally slower and less accurate in detecting words that evoke touch (Connell and Lynott, 2010).

Numerous other behavioural studies highlight the importance of information related to the five modalities in predicting performance in various conceptual tasks such as modality-switching cost paradigms, which shows that checking a property in one modality (e.g., auditory: *rustle* for “leaf”) will take less time after having checked another property in the same modality (e.g., *noise* for “mixer”; e.g., Pecher et al., 2003; Vermeulen et al., 2007; Van Dantzig et al., 2008). Studies have also demonstrated that the performance in lexical and naming decision tasks can also be predicted by perceptual strength (e.g., Connell and Lynott, 2012, 2014; Lynott et al., 2019).

Concreteness and imageability are the traditional variables thought to facilitate word processing (e.g., Paivio, 1969, 1991; Rubin, 1980; de Groot, 1989; Binder et al., 2005). These concepts reflect different aspects of semantic representations. Concreteness refers to the degree to which words refer to people, places and things that can be seen, heard, touched, smelled, or tasted, and imageability is defined to the ease with which it is possible to form a mental image associated with a word (Bonin et al., 2003a). Words processing also appeared to depend on concepts’ age of acquisition (AoA; e.g., the age at which a word was learned) or conceptual familiarity (e.g., the degree to which people come in contact with or think about a concept; e.g., Bonin et al., 2003b; Ferrand et al., 2008; Kuperman et al., 2012; Chedid et al., 2019b). Interestingly, while Vergallito et al. (2020) reported imageability as a stronger predictor of Italian word processing reaction times, studies conducted in other languages (e.g., Canadian French: Chedid et al., 2019a; English: Connell and Lynott, 2012; Dutch: Speed and Brysbaert, 2020) revealed that the effect of perceptual strength was even more important than the effect of well-known psycholinguistic variables, such as concreteness and imageability, suggesting that the importance of these variables could be influenced by cross-linguistic differences. Connell and Lynott (2012) also suggested that imageability and concreteness could, in fact, be biased in favor of the visual modality and would not reflect the sensory richness of a concept (Hargreaves et al., 2012; Newcombe et al., 2012; Yap et al., 2012). This highlights the need to provide an accurate measure of the perceptual basis of concepts by evaluating each modality separately and to investigate the relationships between the perceptual strength and the traditional psycholinguistic characteristics of words.

In a further study, researchers extended their findings about perceptual strength to a new sensory modality, namely interoception, which refers to internal body sensations emanating from the visceral organs (heart, stomach, lungs, and intestines), the autonomic nervous system, and the immune system (Craig, 2002; Craig and Craig, 2009; Critchley and Harrison, 2013; Khalsa and Lapidus, 2016). In this research, Connell et al. (2018) ran a series of exploratory analyses and demonstrated that interoception plays an important role in how individuals experience abstract and concrete concepts, especially emotions. Furthermore, they evidenced that adding the interoceptive strength in the five traditional sensory modalities significantly facilitated words processing in a lexical decision and in a word naming task as measured by shorter reaction times and higher response accuracy. Previous studies have demonstrated close relationships between interoception and depression and anxiety levels (e.g., Schandry, 1981; Harshaw, 2015). Nonetheless, to the best of our knowledge, there is no study specifically investigating the impact of such clinical variables on the interoceptive rating in healthy individuals, which could be meaningful in the investigation of the relationships between clinical states and the perceptual grounding of concepts.

In recent years, perceptual strength norms have been widely developed in different language, e.g., Dutch (Speed and Majid, 2017; Speed and Brysbaert, 2020), Russian (Miklashevsky, 2018), Mandarin (Chen et al., 2019), Italian (Morucci et al., 2019; Vergallito et al., 2020), and Serbian (Đurđević et al., 2016). Despite their essential role in future studies that investigate the interactions between perceptual, conceptual, and semantic processing, the current databases on perceptual experience available in French are (1) those obtained by Chedid et al. (2019a) which only focus on the visual and the auditory modalities and (2) those obtained by Bonin et al. (2015) on the global sensory-motor experience (SERs; Bonin et al., 2015) which did not refer to a specific perceptual modality. Therefore, this paper aims to extend these existing data in French through two original studies presented in the following section. The aims of our first study were (1) to collect modality-specific perceptual strength ratings for 270 words of the French language as well as their respective norms of psycholinguistic semantic variables (concreteness, imageability, conceptual familiarity, and AoA) and (2) to explore the relationship of our newly developed variables with these psycholinguistic ones. As demonstrated for visual and auditory perceptual strength by Chedid et al. (2019a), we hypothesize that the five perceptual modalities would be considered as semantic, through their relationship with the other semantic variables (Juhasz et al., 2011; Connell and Lynott, 2012; Juhasz and Yap, 2013; Bonin et al., 2015). Our second study was designed to collect interoceptive information since it has been shown to participate in the perceptual grounding of conceptual representations (Connell et al., 2018). More specifically, it also aimed to introduce new control variables: the level of anxiety, depression, and the interoceptive awareness of participants, as well as the arousal and the valence of each word. Including these data allowed us to explore important issues in a more clinical perspective as well as determine whether or not these variables define interoceptive strength.

## Study 1: Perceptual Strength Norms for 270 French Words and Their Relationship with Psycholinguistic Variables

### Materials and Methods

#### Participants

One hundred and forty-one French-speaking participants (100 women and 41 men), aged between 18 and 50 (mean age = 25.75; SD = 7.43) with a socio-cultural level of higher education (bachelor’s degree; socio-cultural level = 5.12; five corresponding to the bachelor level; SD = 0.80) took part in this study. They were recruited through social media ads. The inclusion criteria were as follows: Participant must (1) have normal or corrected-to-normal vision, (2) have normal or corrected-to-normal hearing, (3) not have any other sensory disturbances, and (4) do not present any motor disorders. The study was approved by the ethical board of the Psychology and Educational Sciences Faculty of the University of Mons (Mons, Belgium) and followed the guidelines of the Declaration of Helsinki.

#### Stimuli

We selected 270 common nouns of the French language, most of which were concrete nouns. Among these, 188 were selected from Bonin et al. (2003a). The words sample was then completed by 82 common concrete words from the Lexique 3 database (New et al., 2007).

#### Procedure

After completing the consent form and answered questions about personal information, participants completed the rating study on an online platform (Limesurvey). Each participant received the words list and were asked to rate on a Likert-like scale, ranging from 0 (*not at all*) to 5 (*greatly*) the extent to which they experience each word through a specific modality. The modalities were presented in the following order: visual, auditory, haptic, gustatory, and then olfactory. Each question started with an instruction page. The instructions and examples were similar to those from Lynott and Connell (2009, 2013) and from Chedid et al. (2019a) and were as follows: “To what extent, in your opinion, is this word associated with a visual/auditory/haptic/gustative/olfactory sensory experience?” (in French: *« A quel point, ce concept est-il associé, selon vous, à une expérience sensorielle visuelle/auditive/tactile/gustative/olfactive »*; see Appendix A). The ratings were automatically saved by the server in a secured database. Once the participants had completed the first modality, they received an email link to fulfill the second modality and so on. They were informed that they could complete the task in several sessions. The order of the 270 words was randomised for each participant for each question. The entire list was divided, for each modality, into two subsets of words to make it compatible with the Limesurvey format. The subdivision was made according to the alphabetical order of words and the randomisation was made within each subset. A screenshot of an experiment page is reported in Appendix B.

At the end of this assessment, participants were asked to assess, through the exact same online format, the psycholinguistic characteristics of the 270 words, namely concreteness, imageability, conceptual familiarity, and age of acquisition. The instructions for the different variables were either adapted on the basis of original instruction taken from previous published studies (e.g., Bonin et al., 2003a,b; Ferrand et al., 2008; Chedid et al., 2019b) and are available at the Appendix C.

The demographic information of the participants for the separate components of data collection is provided in Table 1.

**Table 1:** Participant demographics for the separate components of data collection.

Component	Sex	Socio-cultural level (M)	Socio-cultural level (SD)	*N*	Age (M)	Age (SD)
Perceptual strength	Female Male	5.15 5.05	0.79 0.81	96 41	26.18 24.43	8.03 5.18
Psycholinguistic variables	Female Male	5.27 5.12	0.78 0.72	80 16	26.96 25.56	8.28 4.72

#### Data Reduction and Analyses

All analyses were conducted using SPSS 21. Significant level was set at *p* = 0.05 throughout analyses. First, we proceeded to a screening of the outliers. The mean and SD of all participant’s ratings in each modality were calculated and participants were discarded from analyses when their mean score fell outside ±2.5 SD from the group mean. The data of five participants (four females) were excluded. The exclusion was done at a participant level in order to keep the same number of participants for all modalities.

For intra and inter-study reliability, Spearman correlation coefficients were computed (since the distribution of our variables did not check the normality assumption) as well as Cronbach’s alphas for inter-item and inter-rater consistency. For the analysis of the relationships between modalities, we ran a principal component analysis (PCA) with an orthogonal (Varimax) rotation. Finally, the relationships between the perceptual strength variables and words’ psycholinguistic characteristics were analysed through correlation analyses.

### Results and Discussion

#### Intra-and Inter-study Reliability

As in Chedid et al. (2019a), we first measured the internal consistency of the ratings. This split-half reliability coefficient is obtained by splitting the ratings of the participants into two groups (according to even and odd participant numbers), and by computing a Spearman correlation between the two groups data for each variable separately. If the ratings of the two groups are well-correlated, they should provide similar results, meaning that the ratings have good internal consistency reliability. The results show significant corrected Spearman correlations for the five perceptual strength (visual: *ρ* = 0.974, *p* <0.001; auditory: *ρ* = 0.949, *p* <0.001; haptic: *ρ* = 0.983, *p* <0.001; gustatory: *ρ* = 0.814, *p* <0.001; and olfactory: *ρ* = 0.916, *p* <0.001).

The Cronbach’s alphas conducted for inter-item consistency showed a high reliability for all modalities (visual: *α* = 0.993; auditory: *α* = 0.986; haptic: *α* = 0.988; gustatory: *α* = 0.975; and olfactory: *α* = 0.986). Participants also showed high inter-rater reliability for each modality, according to Cronbach’s alphas for inter-rater agreement (visual: *α* = 0.985; auditory: *α* = 0.989; haptic: *α* = 0.988; gustatory: *α* = 0.996; and olfactory: *α* = 0.993).

#### Mean Rating and Modality Exclusivity

The overall mean perceptual strength rating for the five modalities are presented in Table 2. As observed across previous studies in different languages (e.g., Lynott and Connell, 2013; Miklashevsky, 2018; Lynott et al., 2019; Speed and Brysbaert, 2020; Vergallito et al., 2020), words were rated as primarily experienced in the visual modality, while the gustatory one was least experienced.

**Table 2:** Mean ratings (M) of perceptual strength (0–5) for 270 words across five modalities, with SD, SE, and 95% CI per scale.

	*M*	*SD*	*SE*	95% CI
Visual	2.66	0.84	0.05	2.56
Auditory	1.09	0.87	0.05	0.99
Haptic	1.86	1.00	0.06	1.74
Gustatory	0.62	1.13	0.07	0.48
Olfactory	0.77	0.96	0.06	0.65

Table 3 shows a sample of the words used in our study with their rating profile across modalities and modality exclusivity scores. As suggested by Lynott and Connell (2009, 2013) and Lynott et al. (2019), the modality exclusivity score represents the extent to which a particular concept is perceived through a single perceptual modality and it is computed by dividing the rating range by the sum, as in the formula below, where *M* is a vector of mean ratings for each of the five perceptual modalities (maxM-minM/Σ M). Each score extends from 0% for completely multimodal concepts, to 100% for completely unimodal concepts. Lynott and Connell (2009) have proposed the following categorisation: 0–35% (low), 35–65% (moderate), and 65–100% (high). For example, the most multidimensional word in our norms is *potato chips* (“chips” in French) with a modality exclusivity of 8.55% because it refers to a concept that can be strongly experimented through the five modalities. Conversely, the word *moon* (“lune” in French) was scored as highly unidimensional with a sensory exclusivity of 88.74% because it refers to a concept exclusively experimented visually. Overall, words had an average modality exclusivity score of 41.31%, which support that our sample of words was experienced in a multimodal way, as it has been demonstrated in previous norming (e.g., Lynott and Connell, 2009, 2013; Winter, 2016; Miklashevsky, 2018; Vergallito et al., 2020). However, our results are conflicting with those observed in the Dutch norms. Indeed, Dutch norms present an average modality exclusivity score slightly higher, with a mean situated at 47% (Speed and Majid, 2017) and 49.7% in a study including a larger panel of words (Speed and Brysbaert, 2020). In particular, Speed and Brysbaert (2020) show that modality exclusivity averages vary according to the category of words. These discrepancies between languages leave open the question of cultural or methodological divergences.

**Table 3:** Sample of words from the norms for a range of modality exclusivity (M.E.) score (%), including their ratings of perceptual strength (0–5) across five modalities.

	Visual	Auditory	Haptic	Gustatory	Olfactory	M.E.	Dominant modality
Alcohol	2.78	0.70	1.98	3.04	2.88	20.59	Gustatory
Candle	3.48	0.58	2.30	0.10	2.70	36.90	Visual
Rooster	2.30	2.77	1.02	0.71	0.69	27.82	Auditory
Star	3.44	0.21	0.38	0.04	0.07	82.26	Visual
Mango	2.35	0.46	2.08	2.74	2.25	23.11	Gustatory
Music	2.02	4.30	0.71	0.04	0.13	59.07	Auditory

Each word was then assigned to a dominant modality according to its strongest perceptual modality (i.e., the one that received the highest mean rating). Table 4 shows the distribution of words over the five modalities, with their mean ratings and modality exclusivity scores. As observed repeatedly across sensory norms (Lynott and Connell, 2009, 2013; van Dantzig et al., 2011; Speed and Majid, 2017; Miklashevsky, 2018; Chen et al., 2019; Morucci et al., 2019; Speed and Brysbaert, 2020; Vergallito et al., 2020), the vast majority of concepts were visually-dominant and the smallest number was words dominated by olfaction. Indeed, similarly to other languages, olfaction appears to be relatively unimportant for the meaning of these words.

**Table 4:** Numbers of words and modality exclusivity (M.E.) scores (as percentage), per dominant modality, with the mean ratings of perceptual strength (0–5) in each modality.

	Dominant modality				
	Visual	Auditory	Haptic	Gustatory	Olfactory
Visual rating	2.61	2.29	2.77	3.12	2.47
Auditory rating	1.05	2.80	1.17	0.69	0.58
Haptic rating	1.76	1.28	2.93	2.51	1.65
Gustatory rating	0.25	0.14	0.70	3.43	1.17
Olfactory rating	0.52	0.55	0.51	2.65	3.23
M.E scores	43.05%	39.55%	35.93%	22.57%	29.09%
*N*	217	13	9	30	1

In line with previous norming ratings (Đurđević et al., 2016; Speed and Majid, 2017; Lynott et al., 2019; Speed and Brysbaert, 2020), visual dominant words had the highest modality exclusivity scores (average of 43.05%) and gustatory modality had the lowest (22.57%), indicating that this is the most multimodal sense. Nevertheless, heterogeneous patterns between norming studies have been observed. For example, for Vergallito et al. (2020), olfaction received the highest exclusivity rating whereas it was the lowest for the study of Lynott and Connell (2013). Chen et al. (2019) showed, for their part, the lowest exclusivity rating for the haptic modality. Once again, differences in methodology, such as item selection, may explain these disparities but a cross-linguistic difference cannot be excluded.

Although they are mostly visual, it was noticed that words with a moderate modal exclusivity score also have moderate perceptual strength scores in the other modalities and can, therefore, be characterised as bimodal (e.g., “sponge” was rated 3.28 for visual and 2.80 for haptic, with negligible presence on the other modalities) or multimodal (e. g., “shopping cart” was rated 2.75 for visual, 1.69 for auditory, and 2.72 for haptic with gustatory and olfactory ratings <1).

Furthermore, the words that were dominant for the gustatory modality had the highest perceptual scores, indicating that the gustatory experience should be perceptually distinct from the other modalities, while scores tend to indicate that, even if most frequent, the visual experience would be the least distinct. These results concerning the highest perceptual scores in the gustatory dominant modality are similar to those obtained in Italian (Vergallito et al., 2020), Dutch (Speed and Brysbaert, 2020), and English (Lynott and Connell, 2013). However, it is possible that the methodology used (e.g., word selection procedure) leads to different results. Indeed, depending on the methodology, different results are observed within the same language. For example, in English, the highest perceptual score is visual in one study (Lynott and Connell, 2009; adjectives) but gustatory in the next (Lynott and Connell, 2013; nouns randomly selected). Similarly, in Dutch, the highest perceptual score shifts from auditory (Speed and Majid, 2017; nouns selected to cover equally all the five modalities) to gustatory (Speed and Brysbaert, 2020; words selected to cover a wide variety of word classes).

The complete norms as well as the appendices may be downloaded from this link https://sharepoint1.umons.ac.be/FR/UNIVERSITE/FACULTES/FPSE/SERVICESEETR/SC_CO/Pages/Appendixes.aspx.

#### Relationship Between Modalities

Relationships between modalities were then examined through PCA with an orthogonal (Varimax) rotation. We used a Kaiser normalization (Kaiser, 1960), with a criterion cut-off score set at 1. The factor solution obtained gave a KMO = 0.611 and a Chi-square in the Bartlett sphericity test of *χ*^2^ (10) = 595.40; *p* <0.001. The five modalities have been reduced to two factors, jointly explaining 77.80% of the original variance. The first with an eigenvalue of 2.51, accounts for 50.27% of the variance and is composed of visual, haptic, gustatory, and olfactory strengths. The second, with an eigenvalue of 1.38, accounts for 27.53% of the variance and is composed only of auditory perceptual strength. The loadings of the dimensions in the two components are shown in Table 5.

**Table 5:** Component matrix obtained from principal component analysis (PCA) with a Varimax rotation.

	Factors	
	1	2
Visual	0.822	0.386
Haptic	0.824	0.332
Gustatory	0.709	−0.583
Olfactory	0.764	−0.493
Auditory	0.272	0.730

The correlation matrix between different modalities is reported in Table 6. The strongest positive correlations were observed between visual and haptic ratings, and between gustatory and olfactory modality. This pattern is evidenced widely in the literature (Lynott and Connell, 2009, 2013; Speed and Majid, 2017; Chen et al., 2019; Lynott et al., 2019; Morucci et al., 2019; Speed and Brysbaert, 2020; Vergallito et al., 2020), suggesting that these associations are replicated across languages. The strongest positive correlation observed between the visual and haptic ratings indicates that words with a strong visual experience are also concepts that could be manipulated (i.e., many things that can be seen can also be touched). The strong correlation observed between the gustatory and olfactory modality, recalling the evidence that concepts that can be tasted (e.g., food) can also be smelled (Mojet et al., 2005). This is not surprising since taste and smell are integrated within the same neural pathway and share overlapping brain networks (de Araujo, 2003; Delwiche and Heffelfinger, 2005; Rolls, 2008).

**Table 6:** Correlation matrix between modalities for mean ratings of perceptual strength.

	Visual	Auditory	Haptic	Gustatory	Olfactory
Visual	–	0.324^∗∗^	0.809^∗∗^	0.361^∗∗^	0.357^∗∗^
Auditory	–	–	0.313^∗∗^	0.050	0.133^∗^
Haptic	–	–	–	0.422^∗∗^	0.423^∗∗^
Gustatory	–	–	–	–	0.668^∗∗^

∗ The Spearman correlation is significant at the 0.05 level.

∗∗ The Spearman correlation is significant at the 0.01 level.

Compared to the other modalities, the auditory modality seems to show singular results across languages. Indeed, whereas visual components tend to cluster with haptic as well as olfactory with gustatory, the auditory modality stands apart from the others. For example, in Russian, their factor analysis shows that auditory modality is not part of any factor and the author concludes that the nature of auditory is unclear and needs further exploration. Likewise, in Mandarin, no clear interaction with the other modalities was found. For our part, the fact that the auditory modality constitutes a factor on its own (see Table 5) seems to confirm that this modality is distinct form the others but requires further investigation.

As can be observed in other languages (e.g., Lynott and Connell, 2013; Speed and Brysbaert, 2020), moderate positive correlations are also observed between haptic-gustatory, haptic-olfactory, visual-gustatory, and visual-olfactory experience, pointing out that things that can be smelled and tasted can also be touched and seen. Interestingly, unlike to the robust pattern observed across different languages and datasets (French: Chedid et al., 2019a; English: Lynott and Connell, 2009; Lynott et al., 2019; Russian: Miklashevsky, 2018; Dutch: Speed and Majid, 2017; Italian: Vergallito et al., 2020), we failed to find any negative correlations on auditory ratings. On the contrary, we observed a positive correlation between auditory-visual and auditory-haptic experiences. Since the 270 words of our study are relatively common, a possible explanation could be that most common concepts for which we have an auditory experience (e.g., the barking dog) can generally be seen and/or touched. This assumption is supported by Chedid et al. (2019a) who suggest that the most common objects (e.g., dog) could be identified through a double visual and auditory association, leading the participants to evaluate it as having a strong perceptual strength. We hypothesize that the differences observed between the French-Canadian norms and our study could be explained by a lower AoA (*M* = 5.92) and a higher concreteness (*M* = 4.54) and books frequency (*M* = 31.06) in the present study, compared to the French-Canadian norms showing instead a higher AoA (*M* = 7.21) and a lower concreteness (*M* = 3.97) and books frequency (*M* = 19.25).

Since Sirois et al. (2006) have found differences on some semantic variables between French-Canadian and European French, it seemed relevant to also question the impact of cultural variables. As we share some words in common with the French-Canadian study (Chedid et al., 2019a), we could question whether there are cultural differences in perceptual strength (visual and auditory modality only available) between Canadian French and European French. We computed means and correlational analyses for 163 concepts in common. Significant positive correlations were found (*p* <0.001) for both visual and auditory modalities. These results suggest that European French speakers rate items in the same way than do French-Canadian subjects. The perceptual strength would, therefore, not seem to be sensitive to a cultural effect.

#### Relationships Between Perceptual Strength and Semantic Variables

Relationships between the five perceptual strength variables and other semantic psycholinguistic variables that are known to affect word processing were also examined (Connell and Lynott, 2012; Juhasz and Yap, 2013; Bonin et al., 2015; Chedid et al., 2019a). These semantic variables included (1) concreteness, which refers to the degree to which words refer to people, places and things that can be seen, heard, touched, smelled, or tasted (Bonin et al., 2003a); (2) imageability, referring to the ease with which it is possible to form a mental image associated with a word (Bonin et al., 2011); (3) conceptual familiarity, reflecting the degree to which people come in contact with or think about a concept (Bonin et al., 2003b); (4) AoA which is the age at which a word was learned, notably collected by asking participants to estimate, in years, the age at which they learned each word (e.g., Ferrand et al., 2008); and (5) objective words frequency (including films frequency and books frequency) defined by the number of occurrences with which the word is encountered in a language (Alario et al., 2004; Bonin, 2007). Excepted objective words frequency taken from Lexique 3 (New et al., 2007), ratings of these subjective variables were collected through the same online questionnaire Limesurvey from 96 participants (80 women and 16 men), aged 18–50 years old (mean age = 26.73; SD = 7.80) with a socio-cultural level of higher education (bachelor’s degree; socio-cultural level = 5.25; SD = 0.77). See Table 7 for a summary statistic for all variables, including words frequency (from New et al., 2007). From this data, we determined the exact number of concrete and abstract words (see Table 8). Ten words with a concreteness rating of less than 3 were judged as abstract (ghost, danger, monster, microbe, vampire, unicorn, war, fever, holiday, and birthday). The following analyses were performed on all words.

**Table 7:** Means and SD, minimums, and maximums for psycholinguistic variables for 270 words.

	M	SD	Minimum	Maximum
Lexical variables (from New et al., 2007)				
Books frequency	31.06	62.43	0.00	461.55
Films frequency	29.31	67.86	0.00	470.30
Semantic variables				
Concreteness	4.54	0.79	2.04	5
Imageability	4.63	0.69	2.44	5
Conceptual familiarity	3.11	1.22	1.32	5
Age of acquisition	5.92	2.01	2.44	9.41

**Table 8:** Mean rating (M) and SD for the five perceptual modality for 270 words divided by category.

		Visual		Auditory		Haptic		Gustatory		Olfactory	
	*N*	*M*	*SD*	*M*	*SD*	*M*	*SD*	*M*	*SD*	*M*	*SD*
Abstract	10	1.31	0.69	0.83	0.75	0.51	0.33	0.11	0.13	0.17	0.16
Concrete	260	2.71	0.80	1.10	0.87	1.91	0.98	0.63	1.14	0.79	0.97

Table 9 shows the results of the Spearman correlation analyses conducted between all variables. We found significant and positive correlations (*p* <0.001) between perceptual strength for each modality and three semantic variables: concreteness, imageability, and conceptual familiarity. These positive correlations indicate that as perceptual strength increased, the values of these semantic variables also increased. This suggests that the more the words are imageable, concrete, and familiar, the higher the perceptual strength is in all modalities. These results extend the data obtained by Chedid et al. (2019a) with the visual and auditory modalities. Miklashevsky (2018) also found positive correlation between imageability and visual, olfactory, gustatory, and haptic modality and suggest that experiences of different nature may contribute to overall imageability of words. For the AoA, we found negative correlations (*p* <0.001), suggesting that the earlier a word is learned, the stronger its perceptual strength appears. These results are also similar to those obtained by Chedid et al. for the visual modality and by Miklashevsky (2018) for visual, olfactory, and haptic ratings. However, as in Miklashevsky (2018), we failed to observe a correlation between AoA and the auditory modality that can be explained by the fact that the auditory modality distinguishes itself from the others, as seen in Table 5.

**Table 9:** Correlation values for the five perceptual strength and the psycholinguistic semantic variables.

	Visual	Auditory	Haptic	Gustatory	Olfactory
Imageability	0.632^∗∗^	0.235^∗∗^	0.555^∗∗^	0.282^∗∗^	0.295^∗∗^
Familiarity	0.857^∗∗^	0.370^∗∗^	0.767^∗∗^	0.356^∗∗^	0.313^∗∗^
Concreteness	0.656^∗∗^	0.298^∗∗^	0.739^∗∗^	0.293^∗∗^	0.289^∗∗^
AOA	−0.250^∗∗^	−0.094	−0.244^∗∗^	−0.188^∗∗^	−0.221^∗∗^
Book frequency	0.333^∗∗^	0.230^∗∗^	0.153^∗^	0.102	0.131^∗^
Film frequency	0.265^∗∗^	0.298^∗∗^	0.072	0.094	0.115

∗ The Spearman correlation is significant at the 0.05 level.

∗∗ The Spearman correlation is significant at the 0.01 level.

For the books frequencies, we observe significant positive correlations with all the variables except the gustatory modality. The film frequency also appeared to be significantly correlated with visual and auditory perceptual strength. These results contrast with those obtained with the Sensory Experience Ratings (Juhasz et al., 2011; Juhasz and Yap, 2013; Bonin et al., 2015) that showed near null (Bonin et al., 2015) to weak (Juhasz et al., 2011) correlations and underline the interest of declining the sensory experience in different modalities as we did. The correlations observed with these semantic variables extend to the five perceptual modalities the initial results made by Chedid et al. (2019a) on the visual and auditory perceptual strength and corroborate the proposition that perceptual strength variables index one aspect of the semantic representations of the words.

It has been shown that visual strength predicts the rate of concreteness and imageability (Connell and Lynott, 2012). Since our words are predominantly visually dominant, we also aimed to test this hypothesis. The results of the linear regression analyses showed that the perceptual strength on all five modalities contributed to the regression model of concreteness ratings (*F*_(5,269)_ = 25.36, *p* <0.001, *R*^2^ = 0.324). More precisely, concreteness can be predicted by a high level of perceptual visual strength (*t* = 3.55; *p* <0.001) and a high level of haptic strength (*t* = 3.50; *p* = 0.001). Unlike data from Connell and Lynott (2012) who only observed visual effects, the tactile variable also contributed to the prediction of the concreteness rating. This difference can be explained by the fact that the vast majority of our concepts are concrete concepts (see Table 8). Therefore, the tactile modality seems to be of great importance. This is corroborated by the study of Speed and Brysbaert (2020) who also observed that visual and haptic rating were the strongest positive predictors for concrete words.

Regarding imageability, only visual perceptual strength contributed to the model (*F*_(5,269)_ = 22,73; *p* = 0.001; *β* = 0.561). So, as expected, imageability can be predicted by high level of perceptual strength. These results are consistent with those of Connell and Lynott (2012) and Speed and Brysbaert (2020). Connell and Lynott (2012) noticed that participants tend to rely on visual experience when generating imageability ratings leading other modalities to be neglected or misinterpreted.

## Study 2: Interoceptive Strength Norms for 270 French Words and its Association with Clinical Variables

It has been shown that interoceptive information participates in the perceptual grounding of conceptual representations (Connell et al., 2018). Our second study was designed to collect interoceptive strength for the same 270 words of our Study 1. Given the literature highlighting the role of the internal state of the individual in the dysfunctions of depression and anxiety (Paulus and Stein, 2010), we introduced new control variables in this study: the level of anxiety, depression, the interoceptive awareness of participants, and the arousal and the valence of each word. We were interested in observing whether these variables would influence the responses for the interoceptive modality which includes sensing the physiological condition of the body (Craig, 2002), as well as the representation of the internal state (Craig and Craig, 2009).

### Materials and Methods

#### Participants and Procedure

Among the 141 participants of the Study 1, 122 (88 women and 34 men), 18–53 years of age (mean age = 26.22; SD = 7.59; socio-cultural level = 5.29; SD = 0.74) accepted to participate to this Study 2. Inclusion/exclusion criteria and procedure were similar as described in Study 1. Participants that agreed to participate to the entire study entered an online contest for a 200€-gift voucher. The procedure follows the same guidelines than those presented in Study 1. For each concept, participants had to rate on a Likert-like scale ranging from 0 (=*not at all*) to 5 (=*greatly*) the extent to which the concept elicit a sensation inside the body (see complete instructions in Appendix D). Sensations refer to the perception of visceral organs (e.g., heart, lungs, and stomach), sensations in the bladder, hunger, thirst, changes of temperature, muscle tensions, pleasure, or pain. Participants were informed that they do not necessarily have to feel all of these sensations at the same time. They were also asked to rate the arousal level of each word on a Likert scale from 0 (*you feel completely calm, lethargic, bored, and/or sleepy*) to 5 (*you feel very stimulated, excited, frantic, and nervous*) as well as the intensity of the valence associated with each word on a scale of −5 (*very negative*) to +5 (*very positive*).

#### Inventories

The anxiety level of the participants was measured with the State Anxiety Inventory of Spielberger (STAI-Y, Spielberger et al., 1983), and the depression level was evaluated through the Beck Depression Inventory (Beck et al., 1996). These two questionnaires were chosen for the reliability of their psychometric properties, in particular their good internal consistencies. The participants also completed the Multidimensional Assessment of Interoceptive Awareness (MAIA, Mehling et al., 2012). This scale is composed of eight sub-scales: (1) *Noticing*: awareness of uncomfortable, comfortable, and neutral body sensations (four questions); (2) *Not-Distracting*: tendency not to ignore or distract oneself from sensations of pain or discomfort (three items); (3) *Not-Worrying*: tendency not to worry or experience emotional distress with sensations of pain or discomfort (three items); (4) *Attention Regulation*: ability to sustain and control attention to body sensations (seven items); (5) *Emotional Awareness*: awareness of the connection between body sensations and emotional states (five items); (6) *Self-Regulation*: ability to regulate distress by attention to body sensations (four items); (7) *Body Listening*: active listening to the body for insight; and (8) *Trusting*: experience of one’s body as safe and trustworthy (three items). These sub-scales present a high reliability, with Cronbach’s alphas between 0.66 and 0.87.

#### Data Reduction and Analyses

All analyses in this study were conducted on SPSS 21. The significant level was set at *p* = 0.05 throughout the analyses. We carried out the same method as in Study 1 to screen the outliers. The data of 18 participants were discarded after this screening, their ratings being 2.5 SD below the mean of the group on the same list. Thus, the data of 104 participants were included in the statistical analyses.

### Results and Discussion

#### Intra- and Inter-Study Reliability

As detailed in Study 1, we first measured the internal consistency of the ratings by calculating the split-half reliability coefficient. The results show significant corrected Spearman correlations for the interoceptive modality, *ρ* = 0.888, *p* <0.001, suggesting that the ratings have a good internal consistency reliability. The reliability shown by the Cronbach’s alpha for inter-items consistency was also good (*α* = 0.991). Participants also showed high inter-rater reliability for this modality, according to Cronbach’s alphas for inter-rater agreement (*α* = 0.974). Results also showed that the reliability for arousal and intensity valence ratings was high, as was shown by the Cronbach’s alpha for inter-items consistency (valence intensity: *α* = 0.984; arousal: *α* = 0.997). Participants also showed high inter-rater reliability according to Cronbach’s alpha for inter-rater agreement (arousal: *α* = 0.950; valence ratings: *α* = 0.990).

#### Mean Rating and Modality Exclusivity

The overall mean ratings of interoception, arousal, and valence as well as the average level of anxiety, depression, and interoceptive awareness of participants are presented in the Table 10.

**Table 10:** Mean rating (M) for Interoception strength, Multidimensional Assessment of Interoceptive Awareness (MAIA), BDI, State Anxiety Inventory of Spielberger (STAI-Y), arousal, and valence with SD, SE, and 95% CI per scale.

	M	SD	SE	CI 95%
Interoception	0.63	0.62	0.038	0.55
MAIA				
Noticing	3.37	0.767	0.142	3.08
Not-distracting	2.70	0.892	0.166	2.36
Not worrying	2.47	1.08	0.202	2.06
Attention regulation	2.58	0.761	0.141	2.29
Emotional awareness	3.27	0.901	0.168	2.92
Self-regulation	2.35	1.18	0.219	1.92
Body listening	2.37	1.23	0.228	1.95
Trusting	3.46	1.33	0.247	2.95
STAI-Y	43.55	9.53	1.77	39.93
BDI	11.79	3.69	0.686	10.39
Arousal rating	2.91	0.286	0.053	2.81
Valence rating	5.48	0.551	0.102	5.27

Table 11 shows a sample of words used in our study with their profile of ratings across modalities and modality exclusivity scores including interoception as a sensory modality. Wilcoxon test conducted to compare the modal exclusivity score with and without the interoception revealed that the final score is significantly modified when interoception is included in the calculation (*p* <0.001). For example, the word “spider” which had a modal exclusivity score of 63.33% (visual unimodal tendency with a score of 3.19 for visual strength and 1.36 for tactile strength) with the five sensory modalities become 46.74% (more multimodal with an interoceptive score of 1.76) when we included interoception in the calculation of modal exclusivity score. This result indicates that interoception contributes to the plurimodality of concepts in agreement with the findings of Connell et al. (2018), suggesting that it is considered as a sixth modality. Since interoception dominates emotional concepts (Connell et al., 2018), we have conducted a complementary analysis to examine whether (1) there is a significant difference between the interoceptive strength of emotional and non-emotional words and (2) whether the modification of plurimodality observed above was different according to the emotionality of the concepts. Table 12 represents mean for interoceptive strength and modality exclusivity associated with emotional and non-emotional concepts. These were categorised according to the participants’ rating of valence; emotional words (*N* = 132) corresponding to those judged negatively (rating between −5 and −2) or positively (rating between +2 and +5) and non-emotional words (*N* = 138) corresponding to those judged neutral (rating between −1 and +1). The Mann Withney analysis conducted on these data revealed a significant difference between emotional and neutral words for the interoceptive rating (*U* = 3090.5; *p* <0.001), confirming that emotional words have a higher interoceptive value, as suggested by Connell et al. (2018). However, Wilcoxon test conducted to compare the modal exclusivity score with and without the interoception showed that the final score is significantly modified when interoception is included in the calculation whether the words are emotional (*p* <0.001) or neutral (*p* <0.001). Thus, although interoception dominates emotional concepts, it contributes to the plurimodality of concepts regardless of their valence. This reinforces its relevance as the sixth perceptual modality in the representation of semantic knowledge.

**Table 11:** Sample of words from the norms for a range modality exclusivity (M.E) score (%), including their ratings of perceptual strength (0–5) across five perceptual modalities and interoceptive modality (0–5).

	V	A	H	G	O	I	M.E	DM
Birthday	2.52	2.55	0.81	0.44	0.31	1.92	26.13	Auditory
Injury	2.49	0.49	2.22	0.32	0.18	2.23	29.12	Visual
Pillow	4.04	1.18	4.08	0.11	1.11	1.34	33.47	Haptic
Smile	4.35	0.60	0.72	0.08	0.09	2.29	52.53	Visual
Thief	1.03	0.42	0.31	0.03	0.11	1.67	41.29	Interoceptive

V, visual; A, auditory; H, haptic; G, gustatory; O, olfactory; I, interoceptive; DM, dominant modality.

**Table 12:** Mean rating (M) and SD for interoception modality and modality exclusivity (M.E) score (%) including interoception as a sixth modality for 270 words divided by category.

		Interoception		M.E.	
	*N*	*M*	*SD*	*M*	*SD*
Emotionnal	132	0.93	0.66	36.03	12.19
Neutral	138	0.32	0.31	38.65	9.01

#### Relationship Between Modalities

Table 13 shows the distribution of words over the six modalities, with their mean ratings and modality exclusivity scores. As shown in Study 1, a large number of concepts were dominated by the visual modality (79.26%) which was followed by the gustatory one (11.11%). Interoception only dominated 2.22% of concepts but, in terms of relative importance, it also dominated the olfactory and haptic modalities. Qualitative analyses conducted on the interoceptive-dominant words revealed that these concepts were those with the lower scores on the concreteness and the imageability scales (e.g., « danger » and « fever »). Accordingly, regression analyses conducted on this issue showed that the interoceptive strength was significantly predicted by low levels of concreteness (*F*_(1,270)_ = 39.46; *p* = 0.001; *R*^2^ = 0.128; *β* = −0.358) and by low levels of imageability (*F*_(1,270)_ = 11.13; *p* = 0.001; *R*^2^ = 0.040; *β* = −0.199). Therefore, these results are in line with a previous study (Connell et al., 2018), showing that concepts that were strongly experienced *via* sensations inside the body tended to be considered as abstract rather than concrete. This pattern of findings suggests that interoceptive strength may capture information of perceptual experience that can hardly be represented by the other modalities. Therefore, as suggested by Connell et al. (2018), interoception offers significant information on concepts that would have otherwise been characterised as lacking of perceptual experience.

**Table 13:** Numbers of words and M.E. scores (as percentage), per dominant modality, with the mean ratings of perceptual strength (0–5) and interoception (0–5) in each modality.

	Dominant modality					
	Visual	Auditory	Haptic	Gustatory	Olfactory	Interoception
Visual	2.63	2.51	3.01	3.12	2.47	1.05
Auditory	1.05	3.02	1.29	0.69	0.58	0.82
Haptic	1.78	1.44	3.14	2.51	1.65	0.56
Gustatory	0.25	0.16	0.77	3.43	1.17	0.07
Olfactory	0.52	0.63	0.55	2.65	3.23	0.12
Interoception	0.50	1.35	0.80	0.88	1.18	1.82
M.E.	39.95%	32.84%	31.86%	22.11%	25.74%	39.24%
*N*	214	11	8	30	1	6

In order to quantify the associations between interoceptive information and the modalities, we ran a PCA with a Varimax (orthogonal) rotation. Our selection of factors was based on both a scree plot of eigenvalues and Kaiser’s criterion (Kaiser, 1960) with the cut-off point set at 1. The factor solution obtained gave a KMO = 0.603, and a Chi-square in the Bartlett sphericity test of *χ*^2^ (15) = 660.54; *p* <0.001, which shows the suitability of the data to perform the PCA. Three components with an eigenvalue greater than 1 were revealed by the PCA and jointly account for 84.90% of the original variance. The first, with an eigenvalue of 2.60, accounts for 43.32% of the variance and is composed by the haptic and visual modalities. The second, with an eigenvalue of 1.39, accounts for 23.09% of the variance and is composed by the gustatory and olfactory modalities. Finally, the third, with an eigenvalue of 1.11, accounts for 18.50% of the variance and is composed by the auditory and interoceptive modalities. The loadings of the dimensions in the three components are shown in Table 14. As can be seen in this table, the modalities are evenly distributed between the three factors. Interestingly, results showed strong associations between the visual and the haptic modalities, as between the gustatory and the olfactory ones. However, the auditory and the interoceptive modalities were considered as distinct factors, despite the significant correlations existing between these variables and the other modalities (see Table 15).

**Table 14:** Component matrix obtained from the PCA with a Varimax rotation.

	Factors		
	1	2	3
Visual	0.893	0.208	0.144
Haptic	0.917	0.220	−0.015
Gustatory	0.164	0.909	0.005
Olfactory	0.231	0.889	0.110
Auditory	0.436	−0.293	0.706
Interoceptive	−0.096	0.288	0.876

**Table 15:** Correlation matrix between modalities for mean ratings of perceptual strength and interoceptive strength.

	Visual	Auditory	Haptic	Gustatory	Olfactory	Interoception
Visual	–	0.324^∗∗^	0.809^∗∗^	0.361^∗∗^	0.357^∗∗^	0.225^∗∗^
Auditory	–	–	0.313^∗∗^	0.050	0.133^∗^	0.184^∗∗^
Haptic	–	–	–	0.422^∗∗^	0.423^∗∗^	0.083
Gustatory	–	–	–	–	0.668^∗∗^	0.280^∗∗^
Olfactory	–	–	–	–	–	0.274^∗∗^

∗ The Spearman correlation is significant at the 0.05 level.

∗∗ The Spearman correlation is significant at the 0.01 level.

Inter-correlations of perceptual strength ratings show that interoceptive experience was relatively associated to other modalities. Indeed, interoceptive experience was positively and significantly related to visual (*ρ* = 0.225; *p* <0.001), auditory (*ρ* = 0.184; *p* = 0.002), gustatory (*ρ* = 0.280; *p* <0.001), and olfactory (*ρ* = 0.274; *p* <0.001) strength. However, it was not correlated with the haptic modality (*ρ* = 0.083; *p* = 0.171). Therefore, the sensations felt inside the body seem to correspond to concepts that can also be seen, heard, smelled, and tasted. It can also be assumed that these sensory modalities are also used to build the physical sensations that a concept generates on the body. The correlations observed here are more important than those observed by Connell et al. (2018). A difference also concerns the haptic modality, for which the results were not significant.

#### Relationships Between Interoceptive Rating and Control Variables

We introduced new control variables: the level of anxiety, depression, the interoceptive awareness of participants, and the arousal and the valence of each word. Indeed, we were interested in observing whether these variables could influence the responses for the interoceptive modality and whether the average scoring for this modality can be predicted by these variables.

Analyses revealed no significant correlations between the inventories scores and interoceptive strength (all values of *p* > 0.05), with the exception of the MAIA “Trusting” subscale, reflecting confidence in one’s body sensations. These results suggest that the more confidence a person has in their body sensations, the higher the rating of the interoceptive strength is. Finally, regression analyses failed to reveal a significant impact of the level of anxiety, depression, and interoceptive awareness predicted on the mean interoception rating (*F*_(10,94)_ = 1.368; *R*^2^ = 0.140; *p* = 0.209).

Spearman’s correlation analyses reveal that valence and arousal ratings were not influenced by participants’ depression and anxiety scores. Regarding interoceptive consciousness, however, we observed a significant negative relationship between “attention regulation” and arousal (*ρ* = −0.227; *p* = 0.020). This suggests that the fewer participants are able to regulate their attention to their body signals for more arousing concepts, the more they strongly “live” the concepts. Results also demonstrated a significant positive relationship between the “not-worrying” scale and concepts valence (*ρ* = 0.462; *p* = 0.009), indicating that the fewer participants worry about their body sensations, the more positively, they will judge the concepts.

In order to determine whether the arousal and valence scores could be predicted by the level of depression, anxiety and interoceptive awareness, we conducted regression analyses. The results were not significant for arousal (*F*_(10,94)_ = 1.23; *R*^2^ = 0.127; *p* = 0.286) and valence (*F*_(10,28)_ = 0.664; *p* = 0.743) in predicting the level of anxiety and depression. We also observed that interoception score is not predicted by either the arousal or the valence (*F*_(2,31)_ = 1.61; *R*^2^ = 0.10; *p* = 0.218). Nevertheless, by dividing the evaluation of emotional and neutral words (see Table 12), we observed that interoceptive strength was significantly predicted by high levels of valence (*F*_(1,104)_ = 7.16; *p* = 0.009; *R*^2^ = 0.057; *β* = 0.258) and by high levels of arousal (*F*_(1,104)_ = 7.74; *p* = 0.006; *R*^2^ = 0.061; *β* = 0.266) for emotional words. This is not the case when considering only the evaluation of neutral words (*F*_(2,104)_ = 1.14; *p* = 0.323; *R*^2^ = 0.003). Yet again, this highlights the importance of emotional aspects in the interoceptive rating.

## General Discussion

This study provided norms for 270 French words for the five classic sensory modalities as well as the interoceptive modality that is based on the perceptual experience of individuals. Psycholinguistic variables ratings (imageability, concreteness, conceptual familiarity, and AoA) were also provided. These norms are freely available from this link https://sharepoint1.umons.ac.be/FR/UNIVERSITE/FACULTES/FPSE/SERVICESEETR/SC_CO/Pages/Appendixes.aspx.

The associations highlighted between the semantic psycholinguistic variables and the five perceptual variables underline the richness of the conceptual representations. The previous results obtained with the French-Canadian norms of visual and auditory perceptual strength (Chedid et al., 2019a) have been extended to the five sensory modalities and corroborate that perceptual strength indexes one of the aspects of the semantic representation of words. Throughout the different analyses, we observed that there were some discrepancies with the norms of other languages. There may be several avenues to explain them. Firstly, these may be related to the selection of items. Indeed, some studies have selected words in order to fairly cover the five modalities (e.g., Speed and Majid, 2017) while others have made a random selection (e.g., Lynott and Connell, 2013) or a selection by word category (e.g., Miklashevsky, 2018). Differences are also observed within the norms of the same language according to the methodology. For example, while the Franco-Canadian norms showed a negative correlation between auditory and visual modality, we showed the opposite pattern. We hypothesize that these differences observed in our words corpus are related to the selection of words that include particularly common and mostly concrete nouns, whereas Chedid et al. (2019a) include both abstract and concrete nouns in their corpus. Secondly, as it has already been argued in the literature (e.g., Vergallito et al., 2020), it is very probable that cross-linguistic differences also explain discrepancies between norms. The existence of potential cross-linguistic and cultural differences in itself justifies the need to develop language-specific norms. Likewise, differences observed within a language [e.g., Lynott and Connell (2009, 2013)] encourage the development of norms that cover different categories of words. Conducting a global study using the same words (divided into categories) across different languages would answer the issue of cross-linguistic and/or methodologic differences.

Our study also provides the first French standards for interoceptive information. To the best of our knowledge, our study test for the first time the role of valence, arousal, anxiety, and depression on interoceptive strength. We showed that the ratings provided by the participants were independent of their level of anxiety, depression, and interoceptive conscience. However, high level of arousal and valence rating has predicted interoception strength and it was the case only for emotional words, highlighting the importance of interoception in emotional content (Connell et al., 2018). Interestingly, we have shown that interoception contributes to the multimodality of concepts, whether emotional or not, which strengthens the proposal of Connell et al. (2018) to consider interoception as a sixth modality.

Interoception also has been shown to be positively associated with visual, auditory, taste, and olfactory perceptual strength, but as a distinct factor from these modalities. A simple explanation can be found in the distinction between interoception, referring to internal stimuli, and exteroception, referring to the perception of the environment by sensory modalities (external stimuli; Craig, 2002). Also, interoceptive concepts were the least concrete and imageable concepts. Our corpus being mainly composed of concrete words, this may explain why this variable is distinct because it is known to have more importance for abstract concepts (Connell et al., 2018).

The methodological limitations that can be pointed out in our study concern the limited number of words included. Also, as the perceptual assessment of the modalities was proposed in a fixed order, we cannot exclude an order effect in the ratings. However, as the evaluation was relatively long, the participants generally completed the questionnaires in several days. Therefore, we believe that this possible order effect would be very limited. Notwithstanding this, our study points to the need to develop standards on a larger scale.

Since the important role of perceptual experience in conceptual representation has been widely evidenced throughout the literature (e.g., Spence et al., 2001; Pecher et al., 2003; González et al., 2006; Marques, 2006; Vermeulen et al., 2007; Kiefer et al., 2008; Fernandino et al., 2016), these norms can serve in the accurate selection of stimuli in linguistic experiments and increase the reliability of the data to better understand how our knowledge is embodied. Future research also could use these norms to investigate the impact of perceptual information in lexical processing. Indeed, the critical role of variables influencing performance can be analysed in tasks such as denomination, lexical, or semantic decision (e.g., Connell and Lynott, 2012, 2014; Lynott et al., 2019; Vergallito et al., 2020). This would provide a better understanding of the key role of perceptual strength in semantics and, more globally, the relationship between perception and the conceptual system.

## Data Availability Statement

The original contributions presented in the study are included in the article/Supplementary Material, further inquiries can be directed to the corresponding authors.

## Ethics Statement

The studies involving human participants were reviewed and approved by the Ethical Board of the Faculty of Pscychology and Educational Sciences of the University of Mons. The patients/participants provided their written informed consent to participate in this study.

## Author Contributions

AM, EW, and IS have the initial ideas. AM and EW developed the experimental material, collected the data, analyzed the data, and prepared the manuscript. IS, LL, and LR reviewed the final versions of the manuscript. All authors contributed to the article and approved the submitted version.

### Conflict of Interest

The authors declare that the research was conducted in the absence of any commercial or financial relationships that could be construed as a potential conflict of interest.
